# Thermostability in endoglucanases is fold-specific

**DOI:** 10.1186/1472-6807-11-10

**Published:** 2011-02-03

**Authors:** Ragothaman M Yennamalli, Andrew J Rader, Jeffrey D Wolt, Taner Z Sen

**Affiliations:** 1 Department of Genetics, Development and Cell Biology, Iowa State University, Ames, IA 50011, USA; 2 Department of Physics, Indiana University-Purdue University Indianapolis, Indianapolis, IN 46202, USA; 3 Biosafety Institute for Genetically Modified Agricultural Products and Department of Agronomy, Iowa State University, Ames, IA 50011, USA; 4 U.S. Department of Agriculture - Agricultural Research Service, Corn Insects and Crop Genomics Research Unit, Ames, IA, 50011, USA; 5 Bioinformatics and Computational Biology Program, Iowa State University, Ames, IA, 50011, USA

## Abstract

**Background:**

Endoglucanases are usually considered to be synergistically involved in the initial stages of cellulose breakdown-an essential step in the bioprocessing of lignocellulosic plant materials into bioethanol. Despite their economic importance, we currently lack a basic understanding of how some endoglucanases can sustain their ability to function at elevated temperatures required for bioprocessing, while others cannot. In this study, we present a detailed comparative analysis of both thermophilic and mesophilic endoglucanases in order to gain insights into origins of thermostability. We analyzed the sequences and structures for sets of endoglucanase proteins drawn from the Carbohydrate-Active enZymes (CAZy) database.

**Results:**

Our results demonstrate that thermophilic endoglucanases and their mesophilic counterparts differ significantly in their amino acid compositions. Strikingly, these compositional differences are specific to protein folds and enzyme families, and lead to differences in intramolecular interactions in a fold-dependent fashion.

**Conclusions:**

Here, we provide fold-specific guidelines to control thermostability in endoglucanases that will aid in making production of biofuels from plant biomass more efficient.

## Background

Understanding the molecular basis of thermostability is essential for protein engineering applications where the thermal stability of a protein could potentially be enhanced. An important industrial application is to genetically engineer plants for increased biofuel production. For example, a thermostable endoglucanase from *Acidothermus cellulolyticus *has been expressed in *Zea mays *subsp. *mays *increasing the hydrolysis efficiency for conversion of plant cellulose to ethanol [1]. Using this transgenic modification allows hydrolysis of cellulose fibers to begin a pre-processing step within the plant. Transgenic corn feedstock expressing thermostable endoglucanases is an innovation that simplifies the hydrolysis of cellulose-derived ethanol, and hence lowers the cost of production. Although the details of the exact mechanism through which cellulases act is far from complete, it is usually considered that endoglucanase generates cellulose chain ends, following an attack by cellobiohydrolase for subsequent hydrolysis [2-4].

Although thermophilic enzymes are industrially important, our understanding of the factors responsible for thermostability in these enzymes is still incomplete. Many studies have addressed this question by comparing homologous protein structures from different families as an aggregate set [5-11] or by focusing on differences for a single family [12-14]. These comparative studies typically analyze several factors such as the energy of unfolding, number of VdW contacts per residue, number of hydrogen bonds per residue, or number of residues involved in secondary structure at the protein level [5]. Often, these studies have sought to identify any detectable relationship between amino acid composition and thermostability. One of the earlier comparative studies with 18 different families of proteins observed that Arg and Tyr are significantly higher in thermophiles, while Cys and Ser are significantly lower in thermophilic proteins [7]. In a study by Sarai's group, the following factors were argued to impart thermostability: Gibbs free energy change of hydration, long-range non-bonded energy, β-strand tendency and average long-range contacts [11]. However, rather than identifying the structural or sequence-based rules governing increased thermostability these comparisons have instead only suggested many physical or structural features that may impart thermostability, including preferences of certain amino acids [7], increased hydrophobicity [8], or even a single amino acid mutation [14]. By extending the comparison to many different pairs of proteins from two organisms (one thermophilic and one mesophilic) factors such as increase in compactness and sequence dependent strong interactions have been identified as two physical mechanisms underlying thermostability [5].

Previously reported studies [5,10,15,16] involving comparison of thermophilic and mesophilic organism's proteome have led to the conclusion that positively charged residues play an important role in imparting thermophilicity. Berezovsky and Shakhnovich [5] concluded that the strategy of organism's adaptation in a thermophilic environment depends on the "evolutionary history" and "sequence-based" mechanism.

In another approach to the proteome level study, the entire proteome of specific organisms were homology modelled from the PDB database and both sequential and structural comparisons were made between thermophiles and mesophiles. Berezovsky et al [15] used two independently different datasets (the Mintz dataset consisting of 2907 protein structures and the Bordner dataset consisting of 435 protein structures). These authors considered a temperature cutoff of 50°C for classifying thermophiles or mesophiles and concluded that positively charged amino acids play a crucial role in thermophilic proteins by stabilizing the interface and overall protein structures. Chakravarty and Varadarajan [16] used 21 mesophilic (900 protein structures) and 9 thermophilic (300 protein structures) organisms as the dataset and a temperature cutoff of 37°C to differentiate thermophiles from mesophiles. Their results showed that Val and Glu are significantly higher in thermophiles and are also solvent exposed. At the same time, Gln, Asn, Ser, Thr, and His are significantly lower in thermophiles. Comparison of intramolecular interactions showed that cation-π interactions are highly significant in imparting thermophilicity. Similarly, Glyakina et al [10] showed that positively charged residues (Lys, Arg and Glu) on the solvent accessible surface are more significant in thermophiles than in mesophiles.

Because the features that cause thermostability for one protein family are not significant for other families of thermophilic proteins, many studies have focused on thermostabilizing features within a single protein family [12,14,17,18]. In the case of the (α/β)_8 _fold in glycosyl hydrolases (GHs), for example, it was reported that a reduction in the number of Gly residues in thermophilic proteins led to greater stability at higher temperatures [12]. However, this study had two shortcomings: the criteria to select a data set of 29 proteins was solely based on higher crystallographic resolution, but not on lower sequence identities, which can bias the results substantially. And among the 29 structures used, there were only three endoglucanase structures (E.C 3.2.1.4) in the thermophilic set whereas none from the same enzyme class in the mesophilic set. Therefore, the study analyzed the lack of Gly preference in thermophilic glycosyl hydrolase enzymes, rather than endoglucanases specifically. In contrast, here we focus specifically on endoglucanases (E.C. 3.2.1.4) that share no more than 70% sequence identity.

From structural comparisons of proteins from a single fold, Sandgren et al experimentally found that a single amino acid mutation of alanine to valine was responsible for thermal stability among the GH12 family of endoglucanases [14]. Although cases where a single residue change imparts increased stability are quite rare, similar examples can be found in a cold shock protein where two residues confer thermostability [19].

The overall inability to identify common trends for thermostability among many different protein families has caused some to speculate that no single rule defines protein thermostability and the factors determining thermostability for one fold or family of proteins may never be universally applicable [12]. More likely, these comparisons between homologous proteins are complicated by the underlying, unknown relationships between protein sequence, structure, and function. For example, several studies implicated intrinsic disorder as a factor in thermostability at low temperatures, where an increase in temperature induces partial, reversible folding of the protein [20,21]. With the recent hypothesis that psychrophilic (cold-loving) proteins are intrinsically disordered [22], this complex relationship is far from resolved. Another issue to consider is the evolution of folds. The emergence of folds within an enzyme family is likely due to convergent evolution of the different protein structures adapting to the same substrate (cellulose for endoglucanases) at different evolutionary periods. Convergent evolution of distinct folds adapting to perform the same function and mechanism is well documented in pathogenic virulence factors [23], lectins [24], toxins [25], receptors [26], and kinases [27]. Hence, the question of the fold effect on thermostability for a protein enzyme family addresses whether convergent evolution for that family adopted different or similar factors to impart thermostability.

Here we investigate how the evolution-driven mechanisms imparting thermostability may vary for different folds. We look at the sequence- and structure-based factors that can contribute to thermostability for the family of endoglucanase proteins across and within three distinct folds, namely the (α/β)_8 _fold, β-jelly roll fold and the (α/α)_6 _fold. We specifically concentrate on 1,4-β-D-glucanases or endoglucanases, which belong to the broader family of enzymes known as glycosyl hydrolases, extracted from bacterial and fungal sources. Glycosyl hydrolases are enzymes that hydrolyze complex carbohydrate moieties and are comprised of cellulase, xylanase, pectinases, β-glucanase, exocellulase, mananase etc [28]. They are widely used in a wide range of industrial applications, such as juice and wine industries for clarification of beverages; feed industries for increasing the digestibility of the feed; paper and pulp industries for pulp bleaching process; textile industries for selective modification of cellulose fibers (depilling); and in reproducing the stonewashing effect on jeans [29].

In the last few years, cellulases have been used in the conversion of biomass to fermentable sugars for ethanol production. Currently they are extracted from microbial sources leading to increased production costs [30]. Considerable efforts are being undertaken towards improved yield and reduced costs of bioethanol production [31]. Even though they share the same structural fold and catalytic mechanism, our knowledge is limited as to why certain endoglucanases are thermophilic. Understanding this limitation is crucial for enhanced utilization of thermophilic endoglucanases for conversion of biomass to bioethanol. Although both protein folds and protein functions play a role in contributing to thermostability, our hypothesis is that protein folds rather than protein families dominate in determining which specific factors are responsible for protein thermostability.

## Results and Discussion

### Fold diversity in endoglucanases

**Figure 1:**
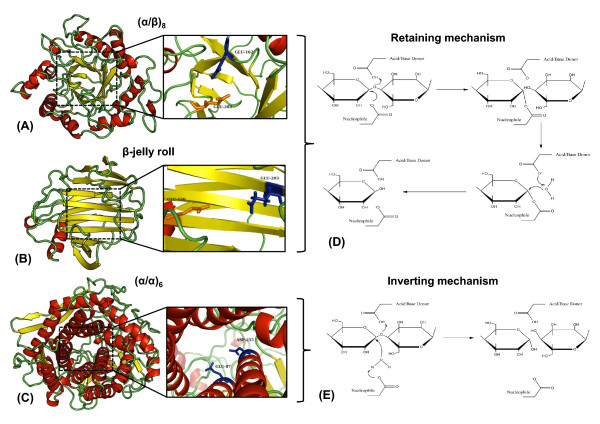
**Different folds in endoglucanases with the catalytic residues highlighted in inset**. (A) (α/β)_8 _fold (*Acidothermus cellulolyticus *pdb id: 1ece) (B) β-jelly roll fold (*Streptomyces lividans *pdb id: 2nlr), and (C) (α/α)_6 _fold (*Clostridium thermocellum F7 *pdb id: 1l1y) are shown in cartoon representation with helices colored in red, sheets colored in yellow, and loops in green. (D) Two-step retaining mechanism of endoglucanase catalysis in (α/β)_8 _and β-jelly roll folds, where the configuration of the anomeric carbon is retained after hydrolysis and a glycosyl-enzyme intermediate is formed. (E) Single-step inverting mechanism of endoglucanase catalysis in (α/α)_6 _fold, where the configuration of the anomeric carbon is inverted; i.e hydrolysis of β-glycosidic bond leads to α-configuration of carbon and vice versa. In (α/β)_8 _and β-jelly roll fold, two glutamic acid residues act as the nucleophile and acid/base donor (shown in Inset (A) and Inset (B)). In (α/α)_6 _fold, a water molecule acts as the nucleophile, and glutamic acid and aspartic acid residues act as the acid/base donor (Inset (C)). The individual residues in stick representation are the nucleophile (in orange) and acid/base donor (in blue).

Endoglucanases (E.C 3.2.1.4) have three distinct structure folds: the (α/β)_8 _fold, β-jelly roll fold and the (α/α)_6 _fold (Figure 1).

#### (α/β)_8 _fold (GH5 and GH44 families)

This fold has an alternating pattern of eight α and β subunits in a single domain, such that the eight parallel β strands on the inside are protected by eight α helices on the outside (Figure 1A). Often referred to as a TIM barrel because it was first discovered in the triosephosphate isomerase (TIM) enzyme, this extremely common fold has been reported to display the highest diversity of enzymatic functions [32]. Endoglucanases in glycosyl hydrolases families 5 (GH 5) and 44 (GH 44) share this fold.

#### β-jelly roll fold (GH7 and GH12 families)

This fold consists of 15 β-strands in two twisted anti-parallel β-sheets, named A and B, that pack against each another (Figure 1B). β-sheet A contains six anti-parallel β-strands forming the back, convex surface while β-sheet B contains nine anti-parallel β-strands arranged to form the front, concave binding surface [29]. Additionally two α-helices pack against the back side of β-sheet B.

#### (α/α)_6 _fold (GH8, GH48 and GH9 families)

The substrate binding cleft in this fold has a tunnel shape formed at the N-termini of six central, parallel α-helices (Figure 1C). These six helices are surrounded by six external α-helices. Unlike the (α/β)_8 _and β-jelly roll folds, the (α/α)_6 _fold utilizes the inverting mechanism for hydrolyzing glycosidic bonds (see below) [33].

### Fold-dependent cellulose hydrolysis in endoglucanases

Cellulose is a linear homo-polysaccharide made up of glucose units that are linked by β-1,4-glycosidic bonds. There are two ends of the polymer: a reducing end, where the terminating anomeric carbon is not linked to another glucose unit, and a non-reducing end. Upon synthesis, cellulose forms as microfibrils that are strengthened by hydrogen, hydrophobic and van der Waals interactions, making it more resistant to hydrolysis than starch, which is made up of α-1,4-glycosidic bonds. Two main different catalytic mechanisms are employed by the glycosyl hydrolases for hydrolysis of glycosidic bonds: the retaining and the inverting mechanisms [29].

#### Retaining mechanism

In this mechanism (Figure 1D), the stereometric configuration of the anomeric carbon is retained in the β-configuration after hydrolysis. A pair of Glu amino acids, separated by 5.5 Å, act as the catalytic residues: one as a nucleophile and the other an acid-base donor. The first step in this double displacement mechanism is glycosylation, where one of the catalytic residue's carboxylic group induces acid-catalyzed leaving group departure simultaneous to a nucleophilic attack on the anomeric carbon to form a glycosyl-enzyme intermediate by the second catalytic residue's carboxylic group. In the second step, a water molecule acts as a nucleophile and the first residue's carboxylic group acts as a base. Once deprotonated, the water molecule is an activated nucleophile that then hydrolyzes the glycosyl-enzyme intermediate leading to a break in the polymer. The (α/β)_8 _(GH5 and GH44 families) and β-jelly roll (GH7 and GH12 families) folds use the retaining mechanism.

#### Inverting mechanism

In this mechanism (Figure 1E), the configuration of the anomeric carbon is inverted; i.e., hydrolysis of β-glycosidic bond leads to α-configuration of carbon and vice versa. The details of this enzymatic mechanism are still not completely known. Glu-Glu pairs usually act as acid-based donors, but recent studies also suggest that Asp may act as the base donor in inverting cellulases [34,35]. Alzari's work [35] suggests specifically Asp as the probable catalytic base in the family GH8. Therefore a pair of Glu-Glu or in some cases Glu-Asp amino acid pairs, separated by 6.5Å to 9.5Å, may act as acid-base donors and a water molecule acts as a nucleophile. Utilizing the water molecule on the opposite side of the sugar ring to stabilize the transition, these residues catalyze the glycosylation or deglycosylation in one step. Unlike the retaining mechanism, this mechanism does not involve the glycosyl-enzyme intermediate. The (α/α)_6 _fold (GH8, GH48 and GH9 families) uses the inverting mechanism for cellulose hydrolysis.

### Amino Acid Composition and Intramolecular Interactions

It has long been suggested that in order to function at higher temperatures, thermophilic proteins contain amino acids that contribute to stronger interactions, which stabilize the structure as compared to mesophiles. Such amino acids form salt-bridges [36], disulfide bonds [37] or cause greater core hydrophobicity [8]. In order to investigate whether this holds true for endoglucanases, we examined the statistical significance of different amino acid compositions and various intramolecular features between mesophilic and thermophilic proteins.

In order to identify the different roles that catalytic mechanism or overall structural fold might play in contributing to these differences, we performed the comparison between thermophilic and mesophilic proteins for each of the following datasets: (A) the dataset comprising all endoglucanases as a enzyme group; (B) two datasets representing each of the two catalytic mechanisms; and (C) three datasets representing the three distinct folds separately. In each of the datasets, the proteins were categorized as thermophilic or mesophilic. Table 1 lists the thermophilic and mesophilic endoglucanases in the three distinct folds.

**Table 1:** Protein sets of Thermophilic and Mesophilic endoglucanases

	***(α/β)***_***8 ***_***fold***			*β-jelly roll fold*			***(α/α)***_***6 ***_***fold***		
	
	GH5 and GH44 families			GH7 and GH12 families			GH8, GH48, and GH9 families		
	
	Organism	PDB id	Length	Organism	PDB id	Length	Organism	PDB id	Length
	
	*Pyrococcus horikoshii*	2zum	458	*Humicola insolens*	1ojj	402	*Clostridium thermocellum NCIB*	1kwf	363
	
	*Acidothermus cellulolyticus*	1ece	358	*Humicola grisea*	1olr	224	*Clostridium thermocellum*	1clc	639
	
*Thermophilic Endoglucanases*	*Clostridium cellulolyticum*	1edg	380	*Rhodotermus marinus*	2bw8	227	*Acyclobacillus acidocaldarius*	3gzk	537
	
	*Clostridium thermocellum*	1cec	343	*Fusarium oxysporum*	3ovw	411	*Clostridium thermocellum F7*	1l1y	678
	
	*Bacillus sp.kas-635*	1g0c	364				*Thermobifida fusca*	1tf4	605
	
	*Thermoascus auranticus*	1h1n	305						
	
	*Uncultured bacterium*	3ii1	535						
	
	*Clostridium thermocellum*	2e4t	509						
	*Prevotella bryantii*	3l55	353	*Streptomyces lividans*	2nlr	234	*Gluconacebacter xylinus*	1wzz	334
	
***Mesophilic Endoglucanases***	*Bacillus agaradhaerens*	7a3h	303	*Hypocrea jecorina*	1oa2	218	*Nasutitermes takasagoensis*	1ks8	433
	
	*Erwinia chrysanthemi*	1egz	291	*Bacillus licheniformis*	2jen	261	*Clostridium cellulolyticum*	1g87	614
	
	*Clostridium acetobutylicum*	3ik2	512	*Aspergillus niger*	1ks5	223	*Clostridium cellulolyticum*	1ia6	441
	
							*Clostridium cellulolyticum*	1g9g	629

A subset of proteins with less than or equal to 70% sequence identity was created as described in the Methods section. A protein is considered thermophilic if the source organism's optimum growth temperature is above 40°C. GH stands for glycosyl hydrolase.

**Figure 2:**
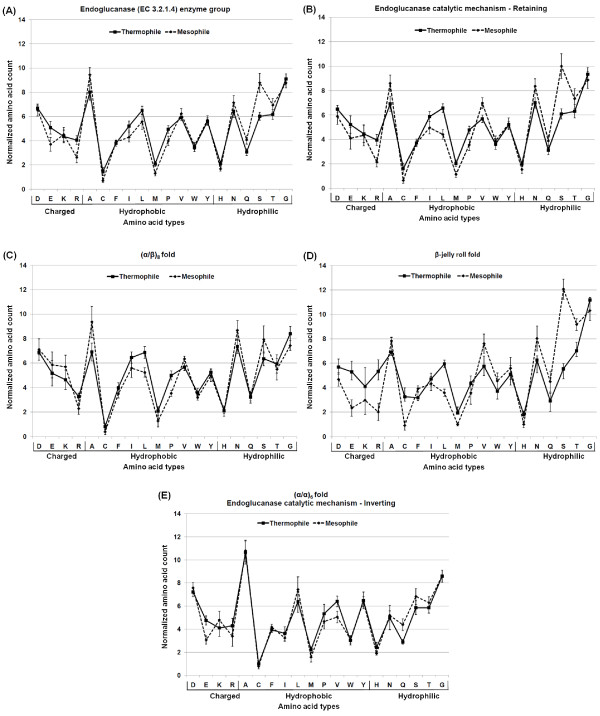
**Normalized amino acid count per protein**. The number of amino acids was normalized with respect to protein length. Amino acid composition of (A) endoglucanases enzyme group, (B) endoglucanase retaining catalytic mechanism, (C) (α/β)_8 _fold, (D) β-jelly roll fold, and (E) (α/α)_6 _fold. The mean of each amino acid and its standard error are shown in each plot, grouped as reported in [5] into charged (DEKR), hydrophobic (ACFILMPVWY), and hydrophilic (HNQST) residues. Solid lines represent thermophilic protein set (squares show means) and dotted lines represent mesophilic protein set (diamonds show means). The statistically significant amino acids are shown in Figure 3. Note that the results for the (α/α)_6 _fold and the endoglucanase inverting mechanism are the same.

**Figure 3:**
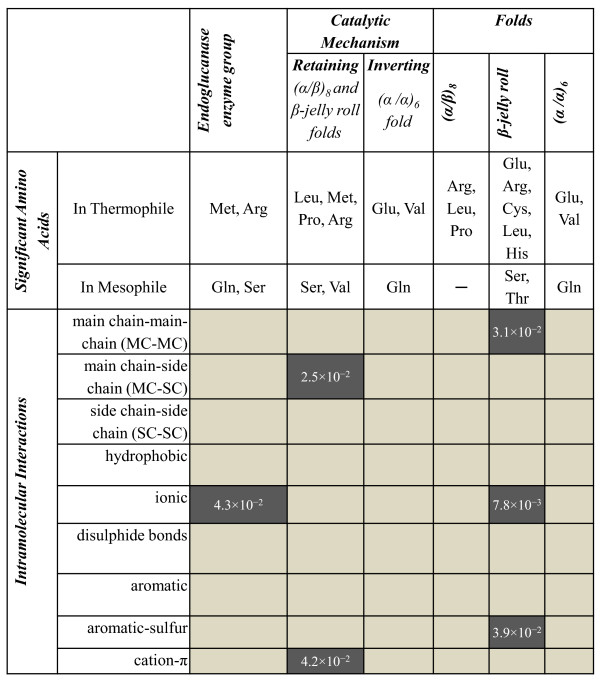
**Statistically significant amino acids and intramolecular interactions between thermophilic and mesophilic endoglucanases**. They are shown in thermophilic endoglucanases as an enzyme group, based on the reaction mechanism, and within each fold, as compared to their mesophilic counterparts (p-value < 5.0 × 10^-^^2^). The statistically significant intramolecular interactions for thermophiles are shaded dark gray. Note that the results for inverting mechanism and on (α/α)_6 _fold are the same.

As described in the Methods section (see below), to identify the significant amino acids (Figure 2 and Figure 3) that make a contribution towards stabilizing the protein structure, intramolecular interactions were calculated. After normalizing with respect the protein length, t-test was performed to identify statistical significance (Figure 3; please see Additional file 1 for detailed quantitative results).

In the larger dataset representing the entire endoglucanase enzyme group, we observed that amino acids Arg and Met are statistically significant among thermophiles, whereas Gln and Ser are statistically significant among mesophiles (Figure 2A and Figure 3). It was previously reported that thermophilic glycosyl hydrolases are significantly missing Gly residues compared to their mesophilic counterparts [8], but we did not observe this negative preference in the case of endoglucanase thermophiles. This difference might be attributed to the fact that endoglucanases form only a small part of the glycosyl hydrolases data set used in the previous study.

We also compared significant intramolecular interactions in thermophiles and mesophiles. For the thermophiles, only ionic interactions were significant, whereas for mesophiles, no intramolecular interactions were significantly different from thermophiles.

We also analyzed the effect of catalytic mechanism on the amino acid distribution and intramolecular interactions. In the retaining mechanism ((α/β)_8 _and β-jelly roll folds) amino acids Leu, Met, Pro, and Arg were significant among thermophiles, whereas Ser and Val were significant among mesophiles (Figure 2B and Figure 3). For the same mechanism, the MC-SC hydrogen bond interactions were significant among thermophiles. In the inverting mechanism ((α/α)_6 _fold) amino acids Glu, Val were significant among thermophiles, whereas only Gln was significant among mesophiles (Figure 2E and Figure 3). However, none of the intramolecular interactions were significant. Strikingly, the significant amino acids and intramolecular interactions are different for the catalytic mechanisms than for endoglucanases as an enzyme group. Another interesting observation is that the significant amino acids and intramolecular interactions for the retaining mechanism are different than those of the individual folds that make up that mechanism. This result strengthens the idea that the analyses of folds provide more informative and detailed understanding of thermostability than a larger set made of many folds.

It is noteworthy that overlaps of amino acid preferences exist if we compare different folds of the endoglucanases. For example, between (α/β)_8 _and β-jelly roll folds, Arg and Leu are significantly higher in thermophiles than in mesophiles (Figure 2C and 2D). Similarly, between β-jelly roll and (α/α)_6 _folds, Glu is significantly higher in thermophiles than in mesophiles (Figure 2D and 2E). Similarities in amino acid preferences within an enzyme class are usually assumed in many protein analyses. These class-specific similarities are actually the basis of those analyses that involve protein properties averaged over many enzyme classes and families. We show here that although similarities exist among the proteins forming an enzyme class, strong fold-specific differences are also present that need to be considered to understand the origin of thermostability.

### Secondary Structure and Solvent Exposure Preference

After identifying a subset of amino acids as statistically significant, we investigated if these amino acids displayed a preference for secondary structure state or solvent exposure.

#### (α/β)_8 _fold

**Figure 4:**
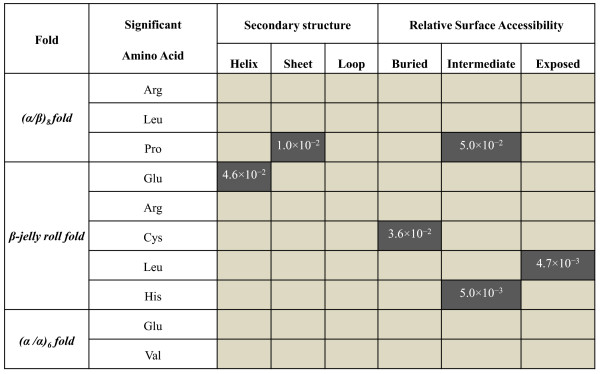
**The p-values for secondary structure and relative surface accessibility preferences for the statistically significant amino acids in thermophilic proteins among the three folds**. The eight state classification of DSSP was reduced into three states (Helix, Sheet, and Loop) as described in the text. The amino acids are classified into three classes according to relative surface accessibility: buried, if less than 9%; intermediate, if between 9-36%; and exposed, if more than 36% [43]. The statistically significant secondary structure and solvent exposure preferences for amino acids are bolded and underlined and p-values are provided. Our analysis showed that the significant amino acids in mesophilic proteins do not show statistically significant preferences for secondary structure or solvent exposure.

In the GH5 and GH44 families, Arg, Leu, and Pro were statistically more significant in thermophiles than in mesophiles, while none of the amino acids were statistically significant among mesophiles (Figure 2C, Figure 3 and Additional file 2). Pro is significantly absent in the β-sheets of the mesophiles whereas prominently present in the β-sheets in thermophiles (Figure 4, Additional file 3 and Additional file 4). Similarly, Pro in thermophiles is significant in the intermediate class of relative surface accessibility (Figure 4).

#### β-jelly roll fold

In GH7 and GH12 families, Glu, Arg, Cys, Leu, and His were statistically significant in thermophiles. The amino acids Ser and Thr were statistically significant in mesophiles (Figure 2D, Figure 3 and Additional file 2). Among the thermophiles Glu was significant in helices but absent in mesophiles (Figure 4, Additional file 3 and Additional file 4). Also all the Cys are significant in the buried class. His is extremely significant in the intermediate class in thermophiles than in mesophiles (Figure 4). Leu is highly significant in the exposed class for thermophiles, as no Leu is exposed in mesophiles.

#### (α/α)_6 _fold

Similarly, in GH8, GH48, and GH9 families, Glu and Val were statistically significant in thermophiles and Gln is statistically significant in mesophiles (Figure 2E, Figure 3 and Additional file 2). Glu and Val were not located in secondary structure and surface accessibility classes in a statistically significant manner (Figure 4, Additional file 3 and Additional file 4).

Among the three folds of endoglucanases, it is interesting to note that the individual amino acid frequencies do not follow a similar pattern. Each fold has specific groups of amino acids that are significant, which cannot be universally applied to other folds, highlighting the importance that sequence plays in determining structure. But also, and more importantly, this result indicates that amino acids responsible for thermophilicity may not rely on enzyme family but more specifically on the protein fold.

### Evolutionarily related thermophilic and mesophilic protein pairs in endoglucanases

**Figure 5:**
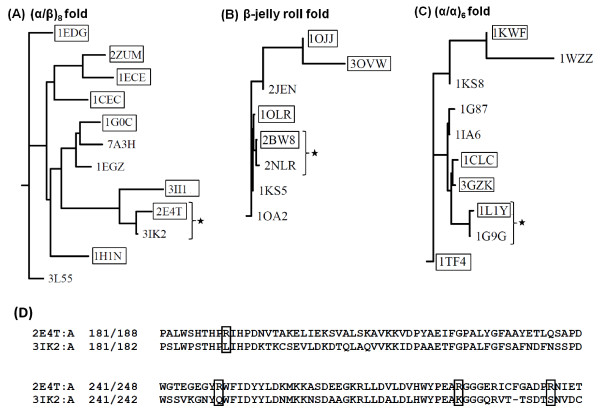
**Phylogenetic tree created using the structure-based multiple sequence alignments in each fold**. The thermophilic proteins are shown in boxes. The thermophilic-mesophilic protein pairs sharing a close evolutionary relationship that have been selected for further analysis are marked with black stars for each of the three folds: (A) (α/β)_8_, (B) β-jelly roll, and (C) (α/α)_6_. (D) A fragment of the pairwise alignment between the thermophilic (pdb id: 2e4t) and mesophilic (pdb id: 3ik2) (α/β)_8 _pair is shown to demonstrate the sequence differences for the statistically significant amino acid arginine in the thermophilic protein. Four arginine positions (shown in boxes) in the thermophilic protein are substituted by different amino acids in the mesophilic protein.

Multiple structural alignment of thermophilic and mesophilic proteins in each fold was performed to identify structurally and evolutionarily similar proteins. Phylogenetic trees were constructed using the structure-based multiple sequence alignments of each fold (Figure 5a, 5b, and 5c). From these trees, the closest pair of thermophilic and mesophilic proteins for each fold was identified and selected for further analysis of possible thermostabilizing differences. Table 2 lists these three pairs of thermophilic and mesophilic proteins: *Clostridium thermocellum *(pdb id: 2e4t) and *Clostridium acetobutylicum *(pdb id: 3ik2); *Rhodotermus marinus *(pdb id: 2bw8) and *Streptomyces lividans *(pdb id: 2nlr); and *Clostridium thermocellum *(pdb id: 1l1y) and *Clostridium cellulolyticum *(pdb id: 1g9g) from the (α/β)_8_, β-jelly roll, and (α/α)_6 _folds respectively.

**Table 2:** Pairwise structural alignment of evolutionarily related thermophilic and mesophilic protein pairs for (α/β)_8_, β-jelly roll, and (α/α)_6 _folds

					Protein length			
							
Fold	Thermophile	Mesophile	RMSD (Å)	Z-score	Thermophile	Mesophile	Sequence identity (%)	Aligned/Gap
**(α/β)**_**8**_	*Clostridium thermocellum* (pdb id: 2e4t)	*Clostridium acetobutylicum* (pdb id: 3ik2)	0.9	8.3	509	512	60.6	507/4

**β-jelly roll**	*Rhodotermus marinus* (pdb id: 2bw8)	*Streptomyces lividans* (pdb id: 2nlr)	1.2	7.2	227	234	33.3	219/9

**(α/α)**_**6**_	*Clostridium thermocellum* (pdb id: 1l1y)	*Clostridium cellulolyticum* (pdb id: 1g9g)	0.8	8.3	678	629	61.6	612/33

Structural superimposition for each of the three pairs shows the RMSD is below 1.5Å and Z-score above 7.2, indicating that the pairwise structural comparison is of a very good quality (Table 2). According to CE algorithm, a Z-score of above 3.5 indicates a very high statistical significance of structural alignment, which can also be seen by the very low number of gaps in each of the alignment (Table 2).

**Figure 6:**
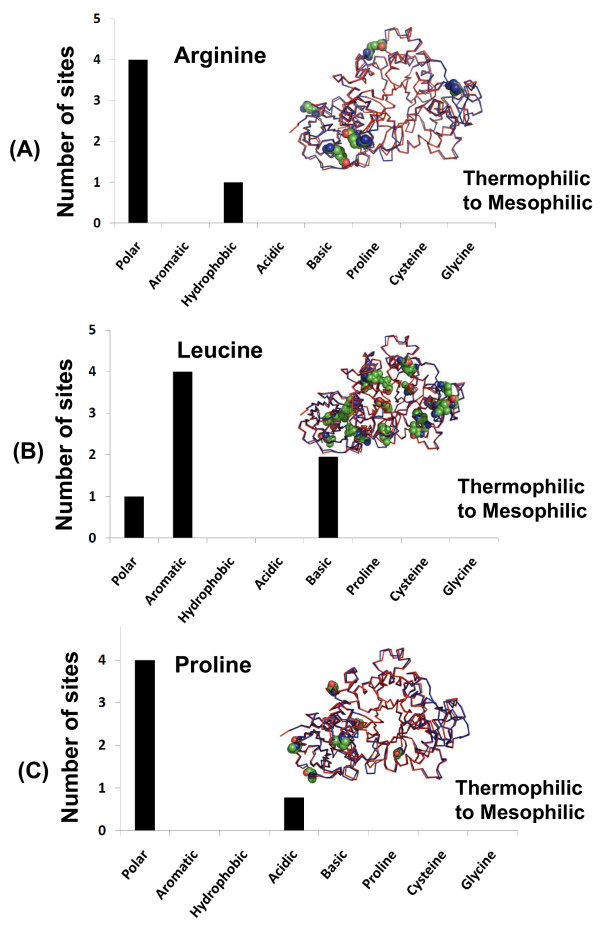
**Mesophilic substitutions at the structurally equivalent positions of significant amino acid types in thermophiles for the (α/β)_8 _fold based on pairwise structural alignments**. Statistically significant amino acid types are (A) arginine, (B) leucine, and (C) proline. Structurally equivalent positions of aligned amino acids between mesophilic endoglucanases and their structurally similar thermophilic counterpart were analyzed for substitutions. The x-axis shows the type or class of amino acids substituted in mesophiles. The count of substitution sites is plotted as a histogram for the following pair shown in Figure 3A: *Clostridium thermocellum *(C^α ^trace colored in red, pdb id: 2e4t) and mesophile *Clostridium acetobutylicum *(C^α ^trace colored in blue, pdb id: 3ik2). Positions of the amino acids are shown in CPK model in superimposed protein structures, where they are differentially colored with respect to atom type.

**Figure 7:**
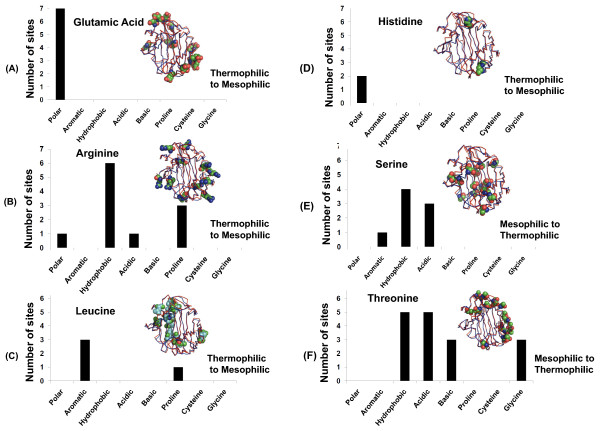
**Substitutions at the structurally equivalent positions of significant amino acid types in thermophiles for the β-jelly roll fold based on pairwise structural alignments**. Statistically significant amino acid types are (A) glutamic acid, (B) arginine, (C) leucine, (D) histidine, (E) serine, and (F) threonine. Structurally equivalent positions of aligned amino acids between mesophilic endoglucanases and their structurally similar thermophilic counterpart were analyzed for substitutions. The x-axis shows the type or class of amino acids substituted in mesophiles ((a)-(d)) and in thermophiles ((e) and (f)). The count of substitution sites is plotted as a histogram for the following pair shown in Figure 3B: *Rhodotermus marinus *(C^α ^trace colored in red, pdb id: 2bw8) and mesophile *Streptomyces lividans *(C^α ^trace colored in blue, pdb id: 2nlr). Positions of the amino acids are shown in CPK model in superimposed protein structures, where they are differentially colored with respect to atom type.

**Figure 8:**
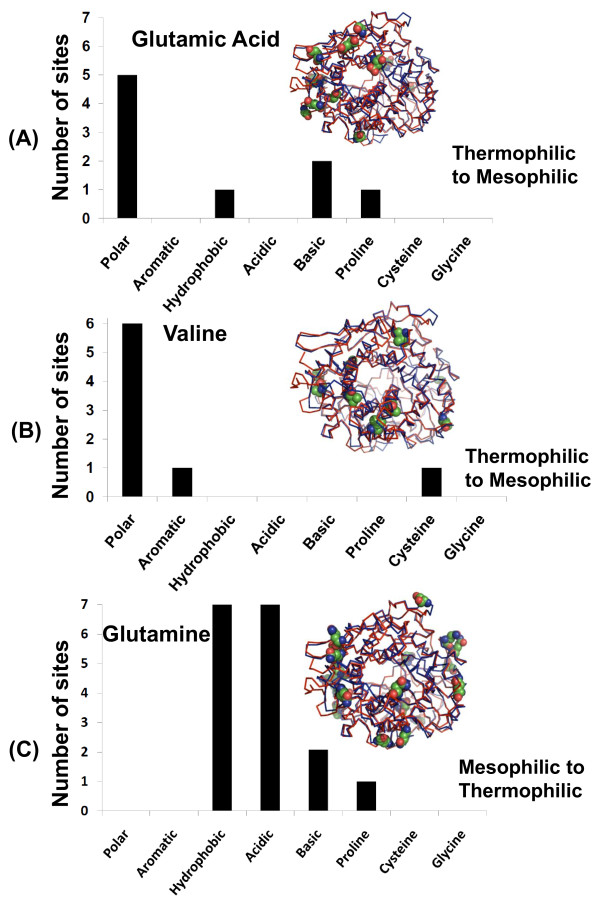
**Substitutions at the structurally equivalent positions of significant amino acid types in thermophiles for the (α/α)_6 _fold based on pairwise structural alignments**. Statistically significant amino acid types are (A) glutamic acid, (B) valine, and (C) glutamine. Structurally equivalent positions of aligned amino acids between mesophilic endoglucanases and their structurally similar thermophilic counterpart were analyzed for substitutions. The x-axis shows the type or class of amino acids substituted in mesophiles ((a) and (b)) and in thermophiles ((c)). The count of substitution sites is plotted as a histogram for the following pair shown in Figure 3C *Clostridium thermocellum *(C^α ^trace colored in red, pdb id: 1l1y) and mesophile *Clostridium cellulolyticum *(C^α ^trace colored in blue, pdb id: 1g9g). Positions of the amino acids are shown in CPK model in superimposed protein structures where they are differentially colored with respect to atom type.

Using pairwise structural alignments for each of these three sets, we were able to identify all the positions where the statistically significant amino acids differ and tally the nature of these substitutions. In particular, each substitution was counted as being polar, aromatic, hydrophobic, acidic, basic, proline, cysteine, or glycine. Figures 6, 7, 8 plot the results for each significant amino acid from each fold. Proline and glycine substitutions were separately counted because the presence of these two amino acids disrupts secondary structure by helix-breaking (Pro) and high flexibility (Gly). Likewise, Cys substitutions were counted separately because of their ability to form disulfide bonds.

For the (α/β)_8 _fold (Figure 6), Arg and Pro (significant in thermophiles) are overwhelmingly replaced by polar amino acids whereas Leu is primarily replaced with aromatic amino acids in the mesophilic counterpart. The replacement of arginines by non-basic polar amino acids in mesophiles supports the view that increased arginine content enhances thermostability. Conversely, the absence of these arginine amino acids leads to a loss of ionic interactions in mesophiles, rendering them enzymatically inactive at higher temperatures.

In the β-jelly roll fold (Figure 7), the amino acids Glu, Arg, and His are substituted with polar, hydrophobic amino acids. Substitution to Pro is higher for Arg indicating the potential for fewer salt-bridges in mesophiles. Quantitatively, we observe only 4 to 11 salt bridges in the mesophiles versus 16 to 40 salt bridges in the thermophiles for this fold. It is unclear as to how the mesophilic protein benefits from substitution of positively charged amino acids with negatively charged amino acids. It is however possible that because some thermophiles lived in acidic environments during their evolutionary history (as some still do), they might have a preference for positively charged amino acids that is carried over the generations that mesophiles do not have a need for. For the Ser and Thr positions (significant among mesophiles) the thermophilic protein has hydrophobic, acidic, and basic amino acids substituted. Interestingly, Thr is substituted with Gly in the thermophilic protein at some positions.

In the (α/α)_6 _fold (Figure 8), Glu and Val are replaced with polar amino acids and to a lesser extent with other amino acid groups. Gln (significant in mesophiles) is substituted to a large extent by hydrophobic, acidic and to a lesser extent with basic amino acid groups in thermophiles indicating that in the thermophilic protein these substitutions contribute towards more intramolecular interactions and extend stability to proteins at higher temperatures such as has been suggested previously as inducing better hydrophobic cores and packing [8].

### Comparison to previous family-level and proteome-level studies

Comparing our results to the previously reported thermophilic-mesophilic comparative studies, we find that observations made for analysis across diverse families of proteins do not necessarily correspond with our family-specific analysis.

For example, Chakravarty and Varadarajan had reported [16] that Val and Glu were significantly higher in thermophiles, which matches only to (α/α)_6 _fold. Also, we find that Glu is significant only in β-jelly roll fold and not in the (α/β)_8 _fold. At the same time, we observe that Gln, Ser, and Thr are significant in mesophiles which is in agreement to their results. However, when it comes to surface accessibility, we do not find any of the significant amino acids in thermophiles as exposed (Figure 4) in the three folds, whereas Chakravarty and Varadarajan found that the significant amino acids in thermophiles are significantly exposed to the surface.

Kumar et al performed a detailed statistical analysis for non-redundant dataset of 18 families [7]. A pair of thermophilic and mesophilic with high structural similarity from each family was selected. Among the amino acids, Arg and Tyr were found to be significantly higher in thermophiles. Among the intramolecular interactions, salt bridges, SC-SC hydrogen bonds were significantly higher in thermophiles.

Comparing our results to Berezovsky et al [15] we also find positively charged amino acids significantly higher in thermophiles, specifically Arg and His. However, we did not find another positively charged amino acid, Lys, significant in thermophiles. Also, since our study involved single-domain proteins we did not calculate as to how the protein interface is stabilized in thermophilic proteins.

Our results also agree with Berezovsky and Shakhnovich's [5] observation about sequence dependent strong interactions for thermostability. However, we see that only the β-jelly roll fold has (MC-MC, Ionic, and Cation-π) significant interactions in thermophiles.

## Conclusions

Understanding the processes responsible for thermostability in endoglucanases is complicated by the large range of structural and sequence diversity these enzymes adopt. Previous studies to derive trends explaining thermostability have focused on large number of protein families, but not necessarily on distinct folds of the same enzyme. In this study we have analyzed the known endoglucanase structures from the PDB and have shown that protein folds rather than protein families are more important when defining rules for thermophilicity. Previous studies [5,7] used the presence or absence of certain types of amino acids in secondary structures (helix, sheet, and loop) as an indication of their role in thermostability. We observed a similar pattern for endoglucanases, as shown in Figure 3, but the types of amino acids contributing to thermostability for the three specific folds studied here differ from those obtained using a more diverse set of proteins. Comparisons between evolutionarily close pairs of thermophilic and mesophilic endoglucanases in each of the fold, reinforces previous assertions that charged amino acids (Arg, His, and Glu) are important for stabilizing the protein at higher temperatures. But one should note that the solvent accessibility of these amino acids also plays a role.

When it comes to thermostability, there is a caveat of applying general heuristic rules based on averaged properties to specific proteins: although thermostability in endoglucanases is usually conferred through altering amino acid composition, in some cases even a single-point mutation is sufficient to convert a mesophilic protein into a thermophilic protein [14].

Upon analyzing the amino acid compositions and intramolecular interactions for the three folds adopted by endoglucanases a paradoxical picture emerges. Namely, although some amino acids are far more significant in thermophiles or mesophiles, they may not significantly alter the overall intramolecular interactions. For example, in the (α/β)_8 _fold, Arg is statistically significant, but ionic interactions are not statistically significant within this fold. Similarly in the β-jelly roll fold, Cys is statistically significant, but the disulfide bridges are not significant. We see a similar pattern for the (α/α)_6 _fold where although Glu is statistically significant, none of the intramolecular interactions are. These results suggest that subtle changes in interactions act as driving factors for thermostability.

For thermophilic proteins, distinct folds have distinct factors that contribute to thermostability, suggesting a fold-specific protein analysis requirement to understand thermostability. Understanding the basis for thermostability aids in engineering enhanced protein activity, which can lead to more cost effective processes for many industrial applications. Our study sheds light on endoglucanases, which could possibly be exploited to increase biofuels crop production by designing a more efficient endoglucanase enzyme. The enzymes currently used in converting biomass to bioethanol for biofuel production have been derived from microorganisms. Unfortunately there are serious technological limitations on biofuel production due to low yield and high production costs for pre-processing enzymes like endoglucanases. Thus the ability to insert a more efficiently designed thermophilic endoglucanase into maize would be very exciting [1].

## Methods

### Protein Dataset

A dataset of endoglucanase protein structures was obtained from the more than 100 glycoside hydrolase (GH) families within the Carbohydrate-Active enZymes database (CAZy) [38]. The enzyme classification number 3.2.1.4 was used to identify all endoglucanases from these various GH families and group them based upon their three-dimensional structural fold. Endoglucanase structures determined by either X-ray crystallography or NMR spectroscopy were classified into one of three structural folds: (α/β)_8_, β-jelly roll, or (α/α)_6_. Using the primary citation of the PDB structure for conformation, each protein was identified as either thermophilic and mesophilic in the following manner: if the source organism's optimum growth temperature (T_L_) is above 40°C, then the protein was classified as thermophilic; if not, as mesophilic. We note that although the melting temperature (T_m_) of a given protein is a better indicator of its thermostability than growth temperature of the organism, the melting temperature is not often widely available. For instance we sought T_m _values for all the proteins in our dataset from the ProTherm database [39], but except for one thermophilic protein (pdb id: 1olr) and for one mesophilic protein (pdb id: 1oa2) the T_m _values are unavailable. Previous studies comparing thermophilic and mesophilic proteins also have mentioned that unavailability of T_m _values is a limiting factor [7]. Since we are only using this information to broadly classify proteins as thermophilic or mesophilic rather than define a direct correlation with T_m_, we follow the precedent of classifying by T_L _[7].

To ensure a similar degree of sequence variation within each of these protein subsets, proteins with greater than 70% sequence and structural similarity were removed using PISCES [40]. For a given set of PDB entries, PISCES uses Combinatorial Extension (CE) algorithm and PSI-BLAST alignments to create a subset of proteins that are evolutionarily related, but with low sequence identity. Our final datasets, listed in Table 1, contained 17 thermophiles and 13 mesophiles.

### Comparisons of sequence and structure based features

Following the results of previous thermophilic-mesophilic comparison studies [7,8], we calculated the statistical significance for several potentially stabilizing features derived from endoglucanase sequences and structures. Statistical significance was defined as having a p-value less than 5.0 × 10^-^^2 ^(95% confidence interval) in an unpaired two-tail t-test using the statistical software R [41] between differences in mesophilic and thermophilic proteins. All the structures in Table 1 are divided into thermophilic and mesophilic structure sets. The amino acid frequency for each type of amino acid was calculated for each protein. These amino acid frequencies were averaged within the family and used as input into the t-tests to determine which, if any, amino acids displayed a statistically significant difference. When an amino acid was observed to be statistically significant, (p < 0.05) then the set, thermophilic or mesophilic, that had a higher mean frequency was determined to be significantly richer in that amino acid. These tests were repeated for three different groupings of thermophilic and mesophilic sets: all endoglucanases, split by their catalytic mechanism, split by their fold. Additional features based upon intramolecular interactions and relative amino acid environment were compared in similar ways.

DSSP [42] was used to determine the secondary structure of proteins. We used the following scheme to translate eight-letter DSSP code into a three-class scheme where secondary structure states of α helix (H), 3-10 helix (G), and π helix (I) are translated into helix; isolated β-bridge (B) and extended strand (E) are translated into sheet; and hydrogen bonded turn (T) and bend (S) are translated into loop. We also used DSSP to obtain a measure of relative solvent accessibility, *A*_*i*_, for each residue, *i*. Equation 1 describes how the relative solvent accessibility is calculated.

(1) $$A_{i}=\frac{{\text{(DSSP solvent accessibility)}}_{i}}{\text{Maximum accessibility for residue of type }i}$$

As suggested previously, this relative solvent accessibility, *A*_*i*_, was classified as: (1) buried if less than 9%, (2) intermediate if between 9-36% and (3) exposed if more than 36% [43]. Another t-test was used to identify statistically significance differences between mesophilic and thermophilic endoglucanases for any amino acids with these various accessibility states. In these tests, the count of each type of amino acid participating in an accessibility state or secondary structure class was normalized by the total number of that particular amino acid type in each protein.

Finally the Protein Interaction Calculator (PIC) [44] was used to calculate the intramolecular interactions, such as hydrophobic interactions; hydrogen bond interactions (main chain-main chain (MC-MC), main chain-side chain (MC-SC), and side chain-side chain (SC-SC)); disulphide bridges; ionic interactions (distance cutoff of 6Å) [45]; aromatic-aromatic interactions (distance cutoff of 4.5Å to 7Å) [46]; aromatic-sulfur interactions (distance cutoff of 5.3Å) [47]; and cation-π interactions (distance cutoff of 6Å) [48]. For statistical analysis, the number of interactions in each category was normalized by the number of residues in each protein.

### Phylogenetic analysis of thermophilic and mesophilic proteins

In order to identify similar regions in evolutionary related thermophiles and mesophiles, we structurally aligned proteins using the multiple structure alignment tool CE [49]. To check the accuracy of alignments we analyzed if the catalytic residues were aligned among the thermophiles and mesophiles. The results were visualized using PyMol [50]. With this simple check for alignment performance, we used the PHYlogeny Inference Package (PHYLIP) [51] to construct a phylogenetic tree of endoglucanases. The structure based multiple sequence alignments were given as input to PHYLIP and distances were calculated using maximum likelihood estimates based on the Jones-Taylor-Thornton matrix [52]. Then, a neighbor-joining method was used to cluster the sequences using the distance matrix. Based on the clustered phylogenetic tree, we identified at least one pair of thermophilic and mesophilic proteins for each fold, that are structurally similar and evolutionarily related.

## Authors' contributions

TZS and JDW conceived the study and participated in its design and coordination and helped to draft the manuscript. RMY conducted the studies and drafted the manuscript. AJR gave valuable suggestions and finalized the manuscript. All authors read and approved the final manuscript.

## Supplementary Material

Additional file 1 **Intramolecular interactions count for the three folds**. Click here for file

Additional file 2 **Results of unpaired t-test**. Click here for file

Additional file 3 **Count of statistically significant amino acids**. Click here for file

Additional file 4 **Results of unpaired t-test**, showing the p-value of statistically significant amino acids (bold and underlined) for secondary structure and relative surface accessibility preferences in thermophiles. Click here for file
