# Supplementary material for: Thermostability in endoglucanases is fold-specific
Source: BMC Struct Biol. 2011 Feb 3;11:10. doi: 10.1186/1472-6807-11-10 (PMC3047435; doi:10.1186/1472-6807-11-10)
Supplement: Additional file 2 — Results of unpaired t-test. [file 1472-6807-11-10-S2.DOC]

**Supplementary Table 1**

**Results of unpaired t-test** showing the p-value of statistically significant amino acids

1. In Thermophiles

| Fold  Amino acid | (α/β)_8_ | β-jelly roll | (α/α)_6_ |
| --- | --- | --- | --- |
|  | GH5 GH44 | GH7 GH12 | GH8 GH9 GH48 |
| D | 7.3×10^-1^ | 2.9×10^-1^ | 5.5×10^-1^ |
| E | 6.8×10^-1^ | **3.5×10^-2^** | **2.3×10^-2^** |
| K | 4.4×10^-1^ | 5.7×10^-1^ | 5.2×10^-1^ |
| R | **9.8×10^-2^** | **2.9×10^-2^** | 4.2×10^-1^ |
| A | 1.0×10^-1^ | 4.9×10^-1^ | 9.0×10^-1^ |
| C | 2.8×10^-1^ | **2.8×10^-2^** | 5.2×10^-1^ |
| F | 4.4×10^-1^ | 1.8×10^-1^ | 5.0×10^-1^ |
| I | 3.2×10^-1^ | 6.2×10^-1^ | 5.6×10^-1^ |
| L | **5.3×10^-2^** | **1.5×10^-3^** | 4.7×10^-1^ |
| M | 2.0×10^-1^ | 1.2×10^-1^ | 2.3×10^-1^ |
| P | **2.8×10^-2^** | 5.1×10^-1^ | 5.3×10^-1^ |
| V | 1.4×10^-1^ | 1.5×10^-1^ | ***8.3*×10^-2^** |
| W | 5.0×10^-1^ | 5.5×10^-1^ | 8.4×10^-1^ |
| Y | 5.9×10^-1^ | 6.7×10^-1^ | 9.0×10^-1^ |
| H | 9.5×10^-1^ | ***8.4*×10^-2^** | 2.7×10^-1^ |
| N | 2.1×10^-1^ | 1.5×10^-1^ | 8.6×10^-1^ |
| Q | 9.5×10^-1^ | 1.9×10^-1^ | 2.6×10^-2^ |
| S | 1.8×10^-1^ | 4.0×10^-4^ | 3.0×10^-1^ |
| T | 7.2×10^-1^ | 4.2×10^-2^ | 5.4×10^-1^ |
| G | 2.9×10^-1^ | 3.5×10^-1^ | 9.4×10^-1^ |

1. In Mesophiles

| Fold  Amino acid | (α/β)_8_ | β-jelly roll | (α/α)_6_ |
| --- | --- | --- | --- |
|  | GH5 GH44 | GH7 GH12 | GH8 GH9 GH48 |
| D | 7.3×10^-1^ | 2.9×10^-1^ | 5.5×10^-1^ |
| E | 6.8×10^-1^ | 3.5×10^-2^ | 2.3×10^-2^ |
| K | 4.4×10^-1^ | 5.7×10^-1^ | 5.2×10^-1^ |
| R | 9.8×10^-2^ | 2.9×10^-2^ | 4.2×10^-1^ |
| A | 1.0×10^-1^ | 4.9×10^-1^ | 9.0×10^-1^ |
| C | 2.8×10^-1^ | 2.8×10^-2^ | 5.2×10^-1^ |
| F | 4.4×10^-1^ | 1.8×10^-1^ | 5.0×10^-1^ |
| I | 3.2×10^-1^ | 6.2×10^-1^ | 5.6×10^-1^ |
| L | 5.3×10^-2^ | 1.5×10^-3^ | 4.7×10^-1^ |
| M | 2.0×10^-1^ | 1.2×10^-1^ | 2.3×10^-1^ |
| P | 2.8×10^-2^ | 5.1×10^-1^ | 5.3×10^-1^ |
| V | 1.4×10^-1^ | 1.5×10^-1^ | *8.3*×10^-2^ |
| W | 5.0×10^-1^ | 5.5×10^-1^ | 8.4×10^-1^ |
| Y | 5.9×10^-1^ | 6.7×10^-1^ | 9.0×10^-1^ |
| H | 9.5×10^-1^ | *8.4*×10^-2^ | 2.7×10^-1^ |
| N | 2.1×10^-1^ | 1.5×10^-1^ | 8.6×10^-1^ |
| Q | 9.5×10^-1^ | 1.9×10^-1^ | **2.6×10^-2^** |
| S | 1.8×10^-1^ | **4.0×10^-4^** | 3.0×10^-1^ |
| T | 7.2×10^-1^ | **4.2×10^-2^** | 5.4×10^-1^ |
| G | 2.9×10^-1^ | 3.5×10^-1^ | 9.4×10^-1^ |
