# Supplementary material for: Thermostability in endoglucanases is fold-specific
Source: BMC Struct Biol. 2011 Feb 3;11:10. doi: 10.1186/1472-6807-11-10 (PMC3047435; doi:10.1186/1472-6807-11-10)
Supplement: Additional file 3 — Count of statistically significant amino acids. [file 1472-6807-11-10-S3.DOC]

**Supplementary Table 2**

**Count of statistically significant amino acids** in three states of secondary structure (helix, sheet, and loop) and relative solvent accessibility (buried, intermediate, and exposed) in each of the three folds

**GH5 GH44**

Arginine

| pdb | helix | sheet | loop | buried | intermediate | exposed |
| --- | --- | --- | --- | --- | --- | --- |
| 2zum | 4 | 3 | 1 | 1 | 5 | 2 |
| 1ece | 4 | 4 | 5 | 5 | 7 | 1 |
| 1edg | 5 | 1 | 7 | 7 | 3 | 3 |
| 1cec | 9 | 1 | 4 | 3 | 10 | 1 |
| 1g0c | 4 | 2 | 5 | 4 | 5 | 2 |
| 1h1n | 4 | 2 |  | 3 | 2 | 1 |
| 3ii1 | 13 | 2 | 8 | 5 | 14 | 4 |
| 2e4t | 3 | 5 | 7 | 4 | 7 | 4 |
|  |  |  |  |  |  |  |
| 3l55 | 2 | 1 | 2 | 2 | 3 |  |
| 7a3h | 5 | 1 | 2 | 1 | 5 | 2 |
| 1egz | 8 | 1 | 1 | 2 | 2 | 6 |
| 3ik2 | 3 | 4 | 1 | 4 | 4 |  |

Leucine

| pdb | helix | sheet | loop | buried | intermediate | exposed |
| --- | --- | --- | --- | --- | --- | --- |
| 2zum | 11 | 6 | 10 | 19 | 6 | 2 |
| 1ece | 11 | 7 | 12 | 20 | 10 |  |
| 1edg | 5 | 5 | 8 | 10 | 5 | 3 |
| 1cec | 15 | 6 | 7 | 22 | 4 | 2 |
| 1g0c | 10 | 8 | 6 | 16 | 5 | 3 |
| 1h1n | 11 |  | 4 | 10 | 5 |  |
| 3ii1 | 14 | 16 | 11 | 25 | 10 | 6 |
| 2e4t | 15 | 10 | 8 | 25 | 6 | 2 |
|  |  |  |  |  |  |  |
| 3l55 | 7 | 5 | 8 | 15 | 4 | 1 |
| 7a3h | 7 | 4 | 5 | 10 | 5 | 1 |
| 1egz | 3 | 6 | 3 | 8 | 3 | 1 |
| 3ik2 | 12 | 9 | 8 | 21 | 6 | 2 |

Proline

| pdb | helix | sheet | loop | buried | intermediate | exposed |
| --- | --- | --- | --- | --- | --- | --- |
| 2zum | 4 | 2 | 18 | 6 | 6 | 12 |
| 1ece | 2 | 2 | 17 | 5 | 5 | 11 |
| 1edg | 3 | 1 | 10 | 7 | 6 | 1 |
| 1cec | 5 | 2 | 4 | 3 | 4 | 4 |
| 1g0c | 6 | 1 | 14 | 6 | 4 | 11 |
| 1h1n | 4 | 2 | 9 | 4 | 4 | 7 |
| 3ii1 | 7 | 4 | 14 | 8 | 8 | 9 |
| 2e4t | 3 | 4 | 19 | 5 | 6 | 15 |
|  |  |  |  |  |  |  |
| 3l55 | 1 |  | 9 | 4 | 3 | 3 |
| 7a3h | 3 |  | 8 | 2 | 1 | 8 |
| 1egz | 3 |  | 7 | 2 | 1 | 7 |
| 3ik2 | 5 | 2 | 14 | 5 | 4 | 12 |

**GH7 GH12**

Glutamic Acid

| pdb | helix | sheet | loop | buried | intermediate | exposed |
| --- | --- | --- | --- | --- | --- | --- |
| 1ojj | 9 | 4 | 16 | 3 | 9 | 17 |
| 1olr | 1 | 3 | 3 | 1 | 3 | 3 |
| 2bw8 | 1 | 8 | 4 | 3 | 1 | 9 |
| 3ovw | 3 | 8 | 9 | 4 | 11 | 5 |
|  |  |  |  |  |  |  |
| 2nlr |  | 4 | 2 | 1 | 2 | 3 |
| 1oa2 |  | 1 | 1 |  | 2 |  |
| 2jen |  | 2 | 2 | 1 | 2 | 1 |
| 1ks5 | 1 | 6 | 2 |  | 4 | 5 |

Arginine

| pdb | helix | sheet | loop | buried | intermediate | exposed |
| --- | --- | --- | --- | --- | --- | --- |
| 1ojj | 3 | 6 | 8 | 1 | 6 | 10 |
| 1olr | 1 | 9 | 6 |  | 7 | 9 |
| 2bw8 |  | 11 | 4 |  | 9 | 6 |
| 3ovw | 3 | 6 | 4 | 1 | 6 | 6 |
|  |  |  |  |  |  |  |
| 2nlr | 1 | 4 | 4 |  | 2 | 7 |
| 1oa2 | 1 | 1 | 1 |  | 3 |  |
| 2jen | 1 | 2 | 1 |  | 2 | 2 |
| 1ks5 |  | 2 |  |  | 1 | 1 |

Cysteine

|  | helix | sheet | loop | buried | intermediate | exposed |
| --- | --- | --- | --- | --- | --- | --- |
| pdb | 3 | 8 | 7 | 16 | 2 |  |
| 1ojj |  | 4 | 1 | 3 | 2 |  |
| 1olr |  | 3 | 1 | 2 |  | 2 |
| 2bw8 | 3 | 7 | 8 | 16 | 2 |  |
| 3ovw |  |  |  |  |  |  |
|  |  |  |  |  |  |  |
| 2nlr |  | 3 | 1 | 2 |  | 2 |
| 1oa2 |  | 1 | 1 | 1 |  | 1 |
| 2jen |  | 1 | 1 |  | 1 | 1 |

Leucine

| pdb | helix | sheet | loop | buried | intermediate | exposed |
| --- | --- | --- | --- | --- | --- | --- |
| 1ojj | 3 | 15 | 7 | 18 | 4 | 3 |
| 1olr | 1 | 10 | 3 | 10 | 3 | 1 |
| 2bw8 | 1 | 9 | 4 | 9 | 3 | 2 |
| 3ovw | 1 | 15 | 4 | 18 | 1 | 1 |
|  |  |  |  |  |  |  |
| 2nlr | 1 | 3 | 3 | 6 | 1 |  |
| 1oa2 | 1 | 5 | 3 | 8 | 1 |  |
| 2jen | 2 | 4 | 1 | 6 | 1 |  |
| 1ks5 | 1 | 7 | 1 | 7 | 2 |  |

Histidine

| pdb | helix | sheet | loop | buried | intermediate | exposed |
| --- | --- | --- | --- | --- | --- | --- |
| 1ojj |  | 5 | 5 | 6 | 1 | 3 |
| 1olr | 1 | 1 | 1 |  | 2 | 1 |
| 2bw8 |  | 2 | 1 | 2 | 1 |  |
| 3ovw | 2 | 4 | 2 | 4 | 3 | 1 |
|  |  |  |  |  |  |  |
| 2nlr |  | 1 |  |  | 1 |  |
| 1oa2 |  | 1 | 1 |  | 2 |  |
| 2jen |  | 3 | 1 | 1 | 3 |  |
| 1ks5 |  | 1 | 1 |  | 2 |  |

Serine

| pdb | helix | sheet | loop | buried | intermediate | exposed |
| --- | --- | --- | --- | --- | --- | --- |
| 1ojj | 5 | 7 | 6 | 9 | 6 | 3 |
| 1olr | 1 | 10 | 2 | 4 | 4 | 5 |
| 2bw8 |  | 8 | 4 | 3 | 3 | 6 |
| 3ovw | 4 | 6 | 16 | 9 | 8 | 9 |
|  |  |  |  |  |  |  |
| 2nlr |  | 15 | 7 | 8 | 5 | 9 |
| 1oa2 | 1 | 18 | 8 | 4 | 10 | 13 |
| 2jen | 1 | 16 | 11 | 6 | 6 | 16 |
| 1ks5 | 4 | 17 | 10 | 4 | 8 | 19 |

Threonine

| pdb | helix | sheet | loop | buried | intermediate | exposed |
| --- | --- | --- | --- | --- | --- | --- |
| 1ojj | 2 | 11 | 15 | 9 | 12 | 7 |
| 1olr | 1 | 10 | 4 | 5 | 5 | 5 |
| 2bw8 |  | 9 | 11 | 3 | 5 | 12 |
| 3ovw | 2 | 9 | 11 | 5 | 11 | 6 |
|  |  |  |  |  |  |  |
| 2nlr | 2 | 11 | 10 | 4 | 4 | 15 |
| 1oa2 |  | 14 | 5 | 3 | 9 | 7 |
| 2jen | 1 | 8 | 10 | 4 | 9 | 6 |
| 1ks5 | 1 | 16 | 4 | 5 | 7 | 9 |

**GH8 GH9 GH48**

Glutamic Acid

| pdb | helix | sheet | loop | buried | intermediate | exposed |
| --- | --- | --- | --- | --- | --- | --- |
| 1kwf | 10 |  | 2 | 1 | 9 | 2 |
| 1clc | 13 | 2 | 13 | 4 | 17 | 7 |
| 3gzk | 15 | 2 | 8 | 5 | 11 | 9 |
| 1l1y | 14 | 6 | 16 | 6 | 20 | 10 |
| 1tf4 | 12 | 4 | 14 | 5 | 16 | 9 |
|  |  |  |  |  |  |  |
| 1wzz | 4 | 1 | 2 |  | 5 | 2 |
| 1ks8 | 9 | 1 | 1 | 5 | 4 | 2 |
| 1g87 | 10 | 3 | 9 | 4 | 14 | 4 |
| 1ia6 | 9 | 1 | 2 | 3 | 6 | 3 |
| 1g9g | 9 | 4 | 13 | 4 | 17 | 5 |

Valine

| pdb | helix | sheet | loop | buried | intermediate | exposed |
| --- | --- | --- | --- | --- | --- | --- |
| 1kwf | 10 | 5 | 5 | 16 | 4 |  |
| 1clc | 12 | 10 | 12 | 24 | 6 | 4 |
| 3gzk | 17 | 13 | 12 | 28 | 11 | 3 |
| 1l1y | 18 | 6 | 11 | 29 | 4 | 2 |
| 1tf4 | 14 | 13 | 15 | 26 | 12 | 4 |
|  |  |  |  |  |  |  |
| 1wzz | 7 | 4 | 7 | 16 | 1 | 1 |
| 1ks8 | 12 | 2 | 7 | 16 | 2 | 3 |
| 1g87 | 13 | 16 | 12 | 22 | 13 | 6 |
| 1ia6 | 7 | 1 | 8 | 12 | 3 | 1 |
| 1g9g | 10 | 7 | 10 | 16 | 6 | 5 |

Glutamine

| pdb | helix | sheet | loop | buried | intermediate | exposed |
| --- | --- | --- | --- | --- | --- | --- |
| 1kwf | 8 |  | 3 |  | 2 | 9 |
| 1clc | 5 | 1 | 7 | 3 | 2 | 8 |
| 3gzk | 2 | 2 | 9 | 3 | 4 | 6 |
| 1l1y | 8 | 2 | 11 | 8 | 4 | 9 |
| 1tf4 | 7 | 4 | 9 | 5 | 4 | 11 |
|  |  |  |  |  |  |  |
| 1wzz | 7 | 1 | 3 |  | 4 | 7 |
| 1ks8 | 10 | 2 | 9 | 5 | 3 | 13 |
| 1g87 | 5 | 2 | 11 | 7 | 2 | 9 |
| 1ia6 | 11 | 1 | 9 | 4 | 3 | 14 |
| 1g9g | 15 | 8 | 13 | 10 | 6 | 20 |
