# Supplementary material for: Thermostability in endoglucanases is fold-specific
Source: BMC Struct Biol. 2011 Feb 3;11:10. doi: 10.1186/1472-6807-11-10 (PMC3047435; doi:10.1186/1472-6807-11-10)
Supplement: Additional file 4 — Results of unpaired t-test, showing the p-value of statistically significant amino acids (bold and underlined) for secondary structure and relative surface accessibility preferences in thermophiles. [file 1472-6807-11-10-S4.DOC]

**Supplementary Table 3**

Results of unpaired t-test, showing the p-value of statistically significant amino acids (bold and underlined)for secondary structure and relative surface accessibility preferences in Thermophiles.

| Fold | Significant Amino Acid | Secondary structure | | | Relative Surface Accessibility | | |
| --- | --- | --- | --- | --- | --- | --- | --- |
|  |  | Helix | Sheet | Loop | Buried | Intermediate | Exposed |
| *(α/β)_8_* | Arg | 3.9×10^−1^ | 9.1×10^−1^ | 3.3×10^−1^ | 8.3×10^−1^ | 8.8×10^−1^ | 7.3×10^−1^ |
|  | Leu | 3.3×10^−1^ | 2.8×10^−1^ | 9.6×10^−1^ | 7.3×10^−1^ | 9.8×10^−1^ | 6.6×10^−1^ |
|  | Pro | 9.1×10^−1^ | **1.0×10^−2^** | 2.4×10^−1^ | 5.5×10^−1^ | **5.0×10^−2^** | 1.8×10^−1^ |
| *β-jelly roll* | Glu | **4.6×10^−2^** | 1.3×10^−1^ | 6.0×10^−1^ | 3.8×10^−1^ | 2.4×10^−1^ | 3.4×10^−1^ |
|  | Arg | 5.5×10^−1^ | 8.1×10^−1^ | 3.8×10^−1^ | 1.4×10^−1^ | 6.0×10^−1^ | 7.3×10^−1^ |
|  | Cys | 1.3×10^−1^ | 4.3×10^−1^ | 9.5×10^−1^ | **3.6×10^−2^** | 8.4×10^−1^ | 2.0×10^−1^ |
|  | Leu | 1.0×10^−1^ | 2.8×10^−1^ | 9.1×10^−1^ | 1.4×10^−1^ | 9.1×10^−1^ | **4.7×10^−3^** |
|  | His | 1.4×10^−1^ | 2.2×10^−1^ | 7.6×10^−1^ | 5.9×10^−2^ | **5.0×10^−3^** | 5.1×10^−2^ |
| *(α /α)_6_* | Glu | 6.8×10^−1^ | 3.5×10^−1^ | 4.1×10^−1^ | 4.8×10^−1^ | 9.6×10^−1^ | 2.0×10^−1^ |
|  | Val | 9.4×10^−1^ | 3.7×10^−1^ | 1.4×10^−1^ | 8.1×10^−1^ | 5.7×10^−1^ | 1.5×10^−1^ |
